# Working towards More Effective Implementation, Dissemination and Scale-Up of Lower-Limb Injury-Prevention Programs: Insights from Community Australian Football Coaches

**DOI:** 10.3390/ijerph15020351

**Published:** 2018-02-16

**Authors:** Angela McGlashan, Glenda Verrinder, Evert Verhagen

**Affiliations:** 1 Department of Community and Allied Health, La Trobe Rural Health School, College of Science, Health and Engineering, La Trobe University, Victoria 3550, Australia; G.Verrinder@latrobe.edu.au; 2 La Trobe Sport and Exercise Medicine Research Centre, La Trobe University, Victoria 3000, Australia; 3 Centre for Sport and Social Impact, La Trobe University, Victoria 3000, Australia;; 4 Amsterdam Collaboration on Health and Safety in Sports, Department of Public and Occupational Health, Amsterdam Public Health Research Institute, VU University Medical Center Amsterdam, 1007 MB. Amsterdam, The Netherlands; e.verhagen@vumc.nl; 5 UCT/MRC Research Unit for Exercise Science and Sports Medicine (ESSM), Department of Human Biology, Faculty of Health Sciences, University of Cape Town, 7701 Cape Town, South Africa; 6 Australian Centre for Research into Injury in Sport and its Prevention, Federation University Australia, Ballarat, Victoria 3350, Australia

**Keywords:** coaching, sport injury prevention, implementation and dissemination, Australian Football, lower-limb injury

## Abstract

Disseminating lower-limb injury-prevention exercise programs (LL-IPEPs) with strategies that effectively reach coaches across sporting environments is a way of preventing lower-limb injuries (LLIs) and ensuring safe and sustainable sport participation. The aim of this study was to explore community-Australian Football (community-AF) coaches’ perspectives on the strategies they believed would enhance the dissemination and scale-up of LL-IPEPs. Using a qualitative multiple case study design, semi-structured interviews with community-AF coaches in Victoria, Australia, were conducted. Overall, coaches believed a range of strategies were important including: coach education, policy drivers, overcoming potential problem areas, a ‘try before you buy approach’, presenting empirical evidence and guidelines for injury-prevention exercise programs (IPEPs), forming strategic collaboration and working in partnership, communication and social marketing, public meetings, development of a coach hotline, and targeted multi-focused approaches. A shift to a culture whereby evidence-based IPEP practices in community-AF will take time, and persistent commitment by all involved in the sport is important. This will support the creation of strategies that will enhance the dissemination and scale-up of LL-IPEPs across community sport environments. The focus of research needs to continue to identify effective, holistic and multi-level interventions to support coaches in preventing LLIs. This could lead to the determination of successful strategies such as behavioural regulation strategies and emotional coping resources to implement LL-IPEPs into didactic curricula and practice. Producing changes in practice will require attention to which strategies are a priority and the most effective.

## 1. Introduction

Lower-limb injuries (LLIs) are an important public health problem among sport participants internationally [1]. Prevention interventions, such as injury-prevention exercise programs (IPEPs), based on identified biomechanical and neuromuscular risk factors, have been developed in order to reduce the risk of LLIs [2]. However, the translation of sport IPEPs into practice has proven difficult [3,4,5], and limited research has been conducted on how to best ensure efficacious, research-tested injury-prevention interventions, including IPEPs, are implemented, disseminated and scaled-up in practice [3,6,7,8,9,10,11,12,13,14,15]. There is a need to understand the practical contexts of sport settings and injury prevention in order to enhance, promote and align IPEPs to practice [9,13,14,16,17].

Wide scale implementation of existing evidence-based lower-limb injury-prevention exercise programs (LL-IPEPs) in community-Australian Football (community-AF) is yet to be fully realized to support the long-term health and wellbeing of players and strengthen the promotion of positive health gains from participations [3,14]. Scaling up is an important process by which health interventions shown to be efficacious on a small scale and/or under controlled conditions are expanded in real world conditions into broader policy or practice [18]. The concept of scaling up is different from routine adoption as it involves an explicit intent to expand the reach of an intervention to new settings or target groups and is accompanied by a systematic strategy to achieve this objective [19]. To bridge this gap in community-AF contexts, one of the first steps in the process is to better understand coaches and athletes (that is, “end users”) perceptions and behaviours regarding injury-prevention programming [7,8,9,14,20]. Coaches play an important role in the development and participation levels of players in community- AF. The coach’s role needs to be considered alongside other advancements in sport injury-prevention research and practice [2,3]. To date, no studies have reported on the perceptions of coaches regarding the implementation, dissemination or scale-up of LL-IPEPs, nor on associated action-planning strategies that might influence coaches on a broader spectrum [3].

Investigating the perceptions and beliefs held by coaches in community-AF is considered relevant to increasing the effectiveness of implementation, dissemination and scale-up of IPEPs for the prevention of LLIs [3,14]. Understanding coaches’ beliefs can contribute to the planning of effective coach IPEPs interventions, and the selection of strategies crucial for successful implementation and sustainability, to improve coaches’ awareness and efficacy in the use of IPEPs, as well as the social and health benefits of community-AF participants [16,21].

This research was undertaken as part of a series of studies that sought to understand the factors associated with adoption and maintenance of LL-IPEPs among community-AF coaches. Specifically, it expands on findings in a previous quantitative study whereby various strategies, including collaboration, feedback/reinforcement approaches, education and other sociocultural themes were recommended by coaches to improve implementation of IPEPs in community-AF settings [22]. Gaining a more in-depth understanding of the views of coaches to facilitate the development of action plans, and promoting multifaceted LL-IPEP interventions to support safe and sustainable participation in community-AF, have been identified as important [2,3,14,16,23]. Exploring perceived strategies to enhance the use of evidence-based LL-IPEP interventions will help to determine the best approaches to assist researchers, sport consultants, coaches and coach educators develop theory and facilitate the application of LL-IPEPs in practice to prevent LLIs, and ongoing participation in sport [24]. The key research question explored was: what are coaches’ insights into strategies that could be used to enhance the planning of LL-IPEP implementation, dissemination and scale-up into community-AF coach practices and settings?

## 2. Methods

### 2.1. Study Design

A qualitative case study design was selected to gain a better understanding of the insights and perceptions of community-AF coaches about strategies that could be utilised to disseminate and scale-up LL-IPEPs into coaching practices [25]. Case study research is a method of empirical enquiry that permits in-depth study of a particular context and social phenomenon that is undertaken in real-life settings [25,26,27]. Case study research is increasingly used as an appropriate and flexible approach to research in sport and coaching sciences (e.g., [28,29]) and other areas in order to comprehensively assess programs, events, activities and processes involving one or more cases, which may or may not be physically co-located with other cases [25,27]. For this study, a collective case study, or multiple case study, design was used. According to Creswell, a multiple case study examines one phenomenon, but multiple cases are used to illustrate the phenomenon. Similarly, Stake [25] notes that a collective case study focuses on one issue as it pertains to multiple cases. Studying multiple cases permits investigation of similarities and differences between cases, which can lead to more robust and reliable evidence, and can contribute to theory building and naturalistic generalisations [25,26,27]. Ethical approval (BO9-083) for this study was granted by the University of Ballarat Human Research Ethics Committee.

### 2.2. Participants

Three male coaches (aged 31 to 35) participated in this study. This sample size is consistent with recommendations in case study research to include 3–4 cases and no more than 4 to 5 [30]. The participants were senior (or head) coaches of adult male community level (i.e., non-elite, grassroots) Australian football teams (Division I and II leagues) in regional and rural Victoria, Australia. Coaches had between 6–10 years of coaching experience. For the purposes of anonymity, participants were allocated pseudonyms.

### 2.3. Procedure

Upon obtaining ethical approval, coaches at five different community-AF clubs were contacted via email and asked to participate in the study. Potential coaches were purposively followed-up and selected from five clubs that were involved in a LL-IPEP clustered randomized controlled trial [31,32,33] aimed at reducing LLIs associated with identified biomechanical and neuromuscular risk factors. Three coaches across three community-AF clubs agreed to participate. All three coaches’ provided verbal and written consent prior to the conduct of the study. Each individual face-to-face interview was arranged and conducted at a mutually convenient time and location (chosen by coaches), including home residence or a workplace meeting room. Interviews, using a semi-structured interview guide, lasted between 60–90 min. The interviews were digitally recorded and later transcribed verbatim by an independent transcriber to ensure a complete and accurate record of the data was obtained. There were no problems encountered in understanding the recordings and completed transcripts (125 single-spaced pages) were checked for accuracy by the first author. Minor edits were made to the transcripts to ensure confidentiality and improve the clarity of statements. The final transcripts and results were sent to coaches for reflection.

### 2.4. Data Analysis and Trustworthiness

Based on recommendations for multiple case study research, the analysis of the data was completed over two stages [27]. Firstly, a within-case analysis was conducted to analyse each of the coach interviews separately. In this stage, the transcribed data for each individual coach interview was descriptively coded and then pattern coded. Next, following the within-case analysis, a cross-case analysis was conducted. Cross-case analysis was undertaken to search for similarities and difference within and between cases. A comprehensive descriptive account was then formulated for each case. Cross-case analysis results will be reported in this study, in accordance with guidelines by Yin [26]. The interview data was stored and managed in Nvivo 10 (QSR International Pty Ltd, Melbourne, Victoria, Australia) [34].

Creswell’s [27] recommendations for case study research were used to ensure trustworthiness and rigor. These were: (1) the triangulation of data sources (individual coach perspectives across different clubs, and layering of data-collection methods—quantitative surveys conducted with community-AF coaches); and, (2) negative case analysis to enhance credibility (all responses were unique and commonly coded). Other strategies [35] included the provision of thick, rich descriptions of coaches’ perceptions and the use of raw quotes to convey findings, conducting a peer review or using a ‘critical friend’ whose role was to review the findings, report back and ask questions. The critical friend was an international sports coach research colleague who was not otherwise involved in the project. Finally, a process of member reflections was conducted.

## 3. Results and Discussion

**Figure 1:**
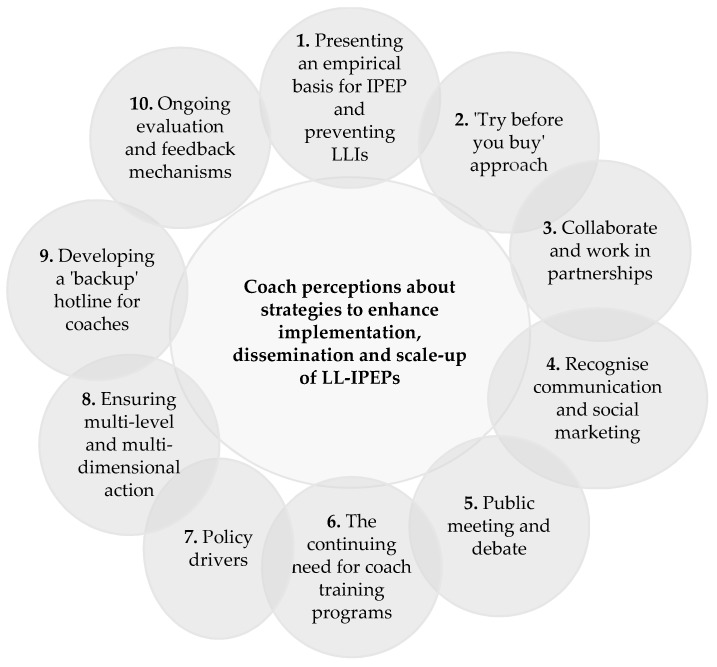
Coach perceptions about strategies to enhance implementation, dissemination and scale-up of LL-IPEPs: lower-limb injury-prevention exercise programs. LLIs: lower-limb injuries.

The coaches perceived a number of cues to action and strategies about how LL-IPEPs could be disseminated and scaled-up to community-AF coaches in order to support safe and sustainable participation agendas (Figure 1). The various strategies identified referred to promoting new ways to address LL-IPEP in community-AF, and what was needed to extend efforts to the wider population of coaches in community-AF.

In general, coaches perceived that it was crucial to present supporting evidence from research efforts (for example, statistics that IPEPs do prevent injury or shows players’ availability to play), remove potential barriers, and consider solutions for coaches to implement IPEPs. Otherwise, they felt that it would be difficult to convince coaches and others to commit to adopting. These approaches were deemed to provide credibility for IPEPs to prevent LLIs. Although discussions surrounding anticipated barriers were not exhaustive, coaches mentioned limited resources, the cost associated with upskilling people to deliver training to coaches and potential others, the cost and purchase of equipment for clubs, development of policy and issues surrounding IPEPs, coach turnover, staff/organisation changes, coach stage of readiness, and the ability to ingrain the program at all levels. Other factors identified were forming strategic collaboration and working in partnership, communication and social marketing, and public meetings, consensus and debate. All three coaches spoke about coach development and learning pathways, both informal and formal, as playing a role in coaches’ readiness to use IPEPs. Finally, the importance of the development of resources such as a coach helpline and ongoing ‘systems’ for IPEPs evaluation and feedback mechanisms were highlighted.

### 3.1. Presenting an Empirical Basis for Injury-Prevention Exercise Programs and Preventing Lower-Limb Injuries Getting the Facts

Presenting an empirical foundation to coaches and important others, rather than ‘intuitive appeal’ for IPEPs’ effectiveness and to prevent LLIs, was seen as an important strategy by all coaches. All coaches commented that the provision of evidence was crucial to convince coaches to adopt IPEPs. Such evidence included well documented data on the injury problem (LLIs) and proposed prevention measures (that is, what worked and how IPEPs prevents LLIs).

One coach provided a scenario that would influence him to use the program:

If a program was presented to me at a seminar, and the one thing I said to … that I asked the … (Victoria Project Manager), initially (prior to the program trial), was ‘is there any statistics yet that it does (LL-IPEP) prevent injury?’ I couldn’t see that it was going to be detrimental to the players’ warm-up, but I think statistical data, which could be provided within a seminar … or a handout that shows … from this program the injury rates were reduced in comparison to X is important. I think figures hit home a lot harder than necessarily saying the proof’s in the pudding … this will change or this will help … has it been proven, and why will it help? … I can certainly see the benefits, and …I think statistics have bigger impact than necessarily a program per se. Somebody sits down and goes wow, they can show that injury prevention in knees, ankles … were reduced by 35 per cent. Why? Because of the training implementation, that also increased players’ attendance on the track, by another rate of 25 per cent, through the maintenance component. The injury-prevention element of it, I think that’s going to sink into potential coaches, or people at the seminar a lot more than the program itself. I’ll go home and analyse the program anyway, but …you also don’t want to leave it to (a coach’s) own interpretation of whether they think it’s going to be beneficial, you need some fairly substantial supporting data with it, which I think, just tops off the presentation. It provides you with a reason, why should you, how come I’m doing this? Now that I know about it, it can only benefit, because these statistics prove. And as I said, you can tailor other stats like you want, but ultimately, there only has to be two, three or four reasons as to why it benefits the playing group, or the players, and coaches will look at it and say, I’ve got to incorporate that.

(Brian)

Similar to other coaches, Geoff was waiting for further evidence to be available. He was motivated to use the evidence and apply this to his coaching and teaching of high-school students:

I’m eagerly awaiting what’s coming out (evidence) because I’ll actually use that as a basis for my teaching. …I teach fitness instructors, I teach Year 12 P.E. (Physical Education). They’re … the people, the next generation, that’s going to use this information. So, from my own professional side of things, I’m waiting for those studies to come down (evidence of study findings to be disseminated). From the coaching side of things, once again, because I can interpret it, I probably will go back out into coaching in some way, shape or form in the next couple of years, so when those studies are there, I’ll probably use it as part of my own periodization throughout the design and warm-ups and cool downs based on it because I do see the benefit at the start and the finish of these programs.

(Geoff)

These examples point to the importance of disseminating credible information on injury to optimizing actions to integrate LL-IPEPs into their coaching practices and other relevant contexts, which reflect previous studies that examined the use of research evidence among coaches [28,36].

### 3.2. ‘Try before You Buy’ Approach

A ‘try before you buy approach’, that is, the degree to which the IPEPs can be experimented with, was perceived as an important strategy by coaches. Being able to try the program was reported as beneficial by coaches and a main reason why they wanted to be a part of the IPEPs trial. They could observe and learn from an innovation and did not appear concerned whether the program had a positive or negative result. It was something that could ultimately develop football and move the game forward in some ways. One coach provided an example where he had benefited from the program. However, he would have also valued the flexibility or option to try out the program in a different football environment:

I had no issues with it at all. I thought it was great. It was well run and everything was fine. It was an opportunity to get new insights. It’s probably tough as well ‘cos I’ve only seen it for that one year so I’ve only really got that to work off (and) if I got to see a comparison … Seeing it run again in a different environment, I could probably have a bit more of an idea of how I could implement it and what might work best in different environments.

(Andrew)

Although Andrew felt the program was ‘great’ and he had ‘no issues with it’, it appears a trial in a different environment may have dispelled uncertainty, and provided further meaning and confidence in his own abilities to implement the IPEP.

In addition to finding out if the program worked under the coaches’ own conditions, or in different conditions or environments, such as with different teams and different clubs, trialing the program may involve reinventing it so it is customized more closely to coaches’ skills, or particular situational-environments [37]. A trial is likely to increase the rate of adoption by coaches, players and significant others associated with football contexts [38]. However, it is also important to ensure that injury outcomes are achieved and any variations coaches make to IPEPs do not have a detrimental effect, and the overall fidelity and benefits of the IPEPs become lost.

### 3.3. Collaborate and Work in Partnership

The coaches considered collaborations, through partnerships across community-AF with coach associations, leagues and clubs as well as input from injury experts, such as researchers, was a strategy for change on a larger scale. Concepts of social support and social networks underpinned this as a strategy and were seen by the coaches as an important mechanism for building and sustaining capacity to prevent and promote LLI interventions.

The emphasis of this collaboration strategy was envisaged to be a ‘top down’ approach, whereby higher-level Australian football systems and approaches could be used to connect injury researchers with other leagues and clubs:

I think there’s ways to get to Football Victoria, the VCFL (Victorian Country Football League), the AFL (Australian Football League), they’re the football bodies that you need to start getting support from, then can deliver that to the leagues, as such, as each league is answerable to their operations. That would be the initial starting point, I think that’s probably the most feasible way to do it, getting leagues together. I think that’s the most achievable, successful way to implement it.

(Brian)

Such a finding reiterates that partnerships are an important vehicle for bringing together diverse material, skills and resources for effective injury prevention outcomes [29]. Partnerships can have an impact on injury prevention by making the best use of different but complementary resources. Collaborations, joint resourcing and planned action can also potentially make a bigger impact on injury outcomes across diverse sectors. As the following indicates, Geoff thought such collaboration together with the formation of partnerships could support resourcing, accreditation pathways and funding, ‘You could approach the AFL development program, investigate resourcing options with them, and also explore professional development points for accreditation and reaccreditation; funds may also be available through this pathway’. (Geoff)

This data highlights a potential synergy leading to more effective solutions than could be achieved otherwise, for example, by an individual or a club working alone. If partnerships are to be successful (establishing, developing and maintaining partnerships), they must have a clear purpose, add value to the work of the partners, and be carefully planned and monitored [29,39,40,41]. As injury prevention specialists continue to work in the implementation sphere, it will be increasingly important to ensure identification of key stakeholders and work with them on solutions that they are willing to apply [11,29,39]. Preparation to support such partners with information and training will be needed [41]. This approach could serve to formulate options and provide recommendations for preventing injury and identifying other issues or concerns.

The emphasis on collaboration and working in partnership appeared particularly relevant to promoting and preventing injury through translating evidence-based IPEPs, which is likely to require extensive work across multiple AF sectors such as clubs, leagues, and associations. ‘Top-down’ versus ‘bottom-up’ approaches will also need to be aligned [42], in addition to considering ‘middle-out’ approaches. There are advantages and disadvantages to top-down, bottom-up and middle-out approaches and all should be explored to ensure effective processes and outcomes are achieved [43,44]. There is an increasing emphasis being made on coalition effectiveness, not merely the formation and maintenance of a broad-based membership [45,46]. This should be considered in context and a partnership tool adapted or developed, and used [47].

### 3.4. Recognising Communication and Social Marketing

One strategy perceived by coaches that holds promise for change is the use of social marketing or media interventions. Social marketing is a framework or structure that draws from other bodies of knowledge such as psychology, sociology and communication theory to understand how to influence peoples’ behaviour [48,49]. Several definitions of social marketing exist, but one of the most useful in this context [50,51] describes social marketing as ‘the application of the commercial marketing technologies to the analysis, planning, execution and evaluation of programs designed to influence the voluntary behaviour of target audiences in order to improve their personal welfare and that of society’ [52] (p. 7). Concepts connected to enhancing health-risk communication, motivating adoption of injury-prevention behaviours, reinforcing preventive messages and potentially sustaining behaviour changes were captured in coaches’ interviews.

Geoff first became aware of the IPEPs through social marketing mechanisms, specifically, a local newspaper and television news report. It was one way that enhanced and reinforced his adopting the program. Other factors included social networks, personal interests and professional development opportunities:

I initially knew about the program from the newspaper and saw it on the local news. I also had contact with a few uni. (University) students who knew what was going on with the program, but also basically through a lot of reading about these types of programs. In the previous year, in one of the clubs where the program had been trialed, I spoke to a few players from there. It was part of professional development for me just reading about what the prevention measures for injuries were, so I was always up to date with the background. I did have awareness of the potential benefits of such programs … I jumped at the chance to be involved.

(Geoff)

Social marketing may be applicable for preparing and stimulating coaches (and others) who may be contemplating change [53]. Geoff’s experience in observing media about the IPEP triggered his interest and he seemed to have no hesitation in later making the decision to agree to trial the program with his team of players in the following season. He was highly intrinsically motivated, ‘I jumped at the chance to be involved’.

(Geoff)

It was also apparent that Geoff’s motivation to adopt the program was also through other means, including discussion within his social networks, that is, players who had been involved in the program the previous season. It is likely that this was also a trigger to find out more and speak to others about it in the football community.

Whilst it is not known whether other coaches observed such media releases, and if indeed they would have the same interest or motivation as Geoff, this approach may have increased awareness and guided the social change of coaches and others (e.g., players, presidents, families and, physiotherapists) to use IPEPs and prevent LLIs. Hence, social marketing through various media has the ability to shape outcomes (knowledge, opinion, attitude, behavior) among individuals, groups, institutions, or communities but also, in turn, be affected by the audience [51]. Further development and testing of such communication strategies seems warranted, as there are some obvious connections to such an approach for adoption of injury prevention programs in community-AF settings, as is the case in other associated areas [51,54,55].

Andrew spoke about elements of social marketing that would help create an environment that was conducive to injury prevention and the uptake of IPEPs. Although Andrew thought it could be personally challenging (‘it’s tough’), to devise such an approach and think about solutions especially as a standalone intervention, he thought ‘key person/s’ were needed to advocate such training programs in marketing approaches. Otherwise, he believed goals of the approach might be ineffective to support normative changes, and supporting coaches to get the best out of their players:

… marketing, yeah, is probably a big one. There’s probably no point–if you don’t have some people to endorse it that are pretty big in it. Even some footballers, you know, well known footballers, that sort of thing, to say that this is the way that football is going, prevention and that sort of thing and if you want the best out of your players.

(Andrew)

This finding is consistent with strategies used in other sports. For example, when the Fédération Internationale de Football Association (FIFA), and the Medical Assessment and Research Centre (F-MARC) disseminated FIFA 11+ they sought the cooperation of famous players and coaches acting as ‘FIFA 11+ ambassadors’ [11,56] which helped significantly in communication with a wide range of coaches [11]. More specifically, within AF, it is often normative to involve players and coaches as ambassadors for various programs and issues [57]. From a theoretical perspective, the finding of the importance of having an ambassador and related data reinforces the application of observational learning (role modelling) [58].

Andrew went on to elaborate on the importance of contextualizing marketing approaches that are personally relevant to coaches. He provided a specific example, focusing on messages relating to coaches’ beliefs (such as notions of team effectiveness - getting the best from their players, having players available, and winning) and aligning these with the prevention of LLIs, as a benefit and an opportunity to achieve this. This is reflected in the following quote:

… our main focus is to have a full list every week to pick from because sides that win Premierships have good depth, they have players to fill spots when players are out. So, if you haven’t got a good side coming up behind you, like in Reserves - If you look back for years, you’ll find that the Reserve sides either played in the Premiership or won it with them. So, if you’ve got those two well balanced, and good sides, good players to be able to fill holes … If you have this side at the start of the year and you hopefully don’t lose anyone by the end of it … if you can keep them on the park and not have any major injuries like broken bones and that sort of thing, you can have a fair crack at the year. So, when you go out and recruit players, you go out and recruit a side. You don’t go out and recruit a side plus extras in case there’s an injury. You go and pick that side that you think is going to win a Premiership.

(Andrew)

Such comments made by Andrew are similar to findings, albeit limited, in other sport injury-prevention dissemination efforts [11]. For example, recently Bizzini, Junge and Dvorak [11] reported some lessons learned during their experience of disseminating injury-prevention programs:

Understanding the coach’s character and highlighting the importance of the program was found to be especially important. Preventing injuries and thereby reducing the number of injured players means that the coach will have more players available for his/her ideal team. Therefore, it is not only information and education about the role of injury prevention that is important, but also speaking the same language as the coach (p. 805).

This reinforces the view outlined by Andrew, in that perceived cost-benefit ratios [59] can tip the balance in favor of the promoted LL-IPEP. It is also likely that social marketing strategies used as cues to prepare coaches in adopting IPEPS must be selected to correspond with coaches’ preferences and information-processing styles [51]. Andrew reiterates this by commenting on the importance of preparing social marketing and informative material by using statistics with visual appeal:

Probably the information’s always a big one … We’re pretty visual and we like to see some results before taking it on … So, if we had some good stats (statistics) on that side of things … you know … if there is some time spent on creating material it’s actually ‘gonna’ work, and continue to send information out there over time. That would be a big thing.

(Andrew)

This finding further reinforces notions of ensuring information and statistics that the program works will help support efforts, or assist in sustaining behavioral changes over time. Promotions could be tailored using different mediums, and could include: the weekly footy program, signage around the ground, billboards, electronic/scoreboards, posters/infographics, league-based radio, club and AFL websites, social media (for example, Facebook, Twitter, and other apps) and other components or events. There are examples within AF such as the use of iPads, advertising on signage around the ground, and events. These could be adapted and evaluated along with sport-injury prevention [60] and other areas [51,54].

While the coaches’ comments do not capture the full extent of what social marketing can offer at present, appropriate communication and social marketing strategies can be used as a systematic approach to understand and strategically respond to coaches’ characters and the context surrounding injury-prevention behavioral domains. Overall, coaches’ comments offer some guidelines for communication planning that can be extended in future [49,61]. In addition, they can be applied within strategic frameworks and linked to key theories of health communication and health behavior for optimizing effective research and practice relevant to social marketing [61].

### 3.5. Public Meeting and Debate

Public meeting and debate was a further strategy discussed by coaches. This strategy was believed to be particularly important for scale-up of IPEPs to target a broad range of coaches and significant others involved in community-AF environments. For example, coaches mentioned that by including public meetings, it would not only support them to embed programs such as IPEPs into clubs, but it would also save them time and effort in convincing others about the credibility of the program. Indeed, public meetings can bring diverse groups of stakeholders together for a specific purpose and have numerous advantages [62]. The main advantages can include: (1) introducing a project or issue to the community; (2) diversifying information-sharing and providing all participants a chance to voice their concerns, issues or ideas; (3) disseminating detailed information and decisions throughout the football community; (4) providing opportunities for exploring alternative strategies and building consensus, including creating consensus for action on any complex issues (for example, other health and safety concerns or priorities) that might require broad-based community input [62].

Brian, for example, reinforced the use of public meetings and particularly commented about the importance of identifying key stakeholders at football clubs:

The easiest way to deliver it would be to hold a general (or public) meeting, and you’d need to deliver it to coaches, presidents, football managers … get all the key people at the club/s. You can’t invite whole committees to a meeting as such, but you can get the president, the football manager, all coaches, from the U18s, seniors, reserves and juniors—if you’re wanting to go through junior clubs.

(Brian)

This finding underlines lessons learned from other sports and implementing large scale injury prevention programs suggests that identifying key stakeholders (often coaches) is an important step in enhancing the success of preventive initiatives [7,12]. As a sport injury-prevention specialist, identifying the key stakeholders and working with them on solutions that they are willing to apply is important [7,12]. Being prepared to support these partners with information and training is vital [7,12].

Coaches further recommended that football governance boundaries be considered when organising public meetings/seminars and to support promotion actions. This approach was suggested to overcome any potential logistical barriers (for example, attendance, time or travel concerns) for stakeholders. For example, one coach commented that formally affiliated leagues could be invited to a seminar:

The easiest way is to get it in a seminar environment, so if you’re talking about the Ballarat Football League as an example–they’re affiliated with three leagues–the Ballarat Football League, the Central Highlands League, and the Maryborough Football League. Not all leagues are fortunate enough to have the facility (governance) that they control all three.

(Brian)

Not only were the governance boundaries important and/or perceived to be one of the easiest ways to plan a series of public meetings to promote IPEPs, the delivery of the information presented was deemed important. Brian spoke of his initial ideas in relation to the format of public meetings/seminars. He suggested presentations and handouts about IPEPs and interrelated topic areas could be useful, in addition to providing coaches and other key actors with a comprehensive IPEP package/guidebook resource about the program and how it could be effectively implemented into clubs:

In a large meeting/seminar you could get representatives to present programs (IPEPs) and information on related topics. Handouts could be provided about the program. You could go through the information and have the program packaged to take away, it would have probably been enough for me to then go away and implement it.

(Brian)

This finding demonstrates that Brian recognized the value of public meetings and that a ‘methods and material orientation’ to IPEPs’ implementation may be suitable for some coaches, including himself, to transfer into their coaching practices and football environments. However, he was also cognizant that this approach may not be as effective or conducive to promote IPEPs to a range of stakeholders in such a setting. He stressed the importance of ensuring any public meeting is not promoted as a ‘one size fits all’:

The program (IPEP), when it was presented to me, provided the resources of two girls (IPEP trainers) who were going to come and run the program. So there are different ways that it could be delivered and this strategy could be discussed (e.g., direct or indirect approach) … I think that it’s got to be a fairly in-depth seminar that talks about each stage (of IPEP integration, delivery and maintenance in the long term) so that people in the seminar understand. Some people that are football coaches, they’re going to have a better understanding of the … or jargon, with regards to what we are talking about, with exercise or limbs or stretches or implementation of what your trying to do, than compared to … maybe someone who may still have even a level one coaching accreditation. So, it’s having that sort of open forum, and delivering it to everyone, you can’t send everyone from every football club, it’s not feasible, and it’s not achievable. You’ve got to hope that the information delivered is attainable and explainable, for the people that take it away, or the environment is open enough that people feel comfortable to ask the questions that are necessary. At least have a ‘Q and A’ afterwards, where those embarrassed, or quieter people can come up to one or two people who are doing the seminar to do so.

(Brian)

It is likely, based on the notions discussed by Brian that identifying the appropriate mode and content of delivery of sport-injury prevention at public meetings will need to be explored to help shape and standardize education of IPEP interventions. This is important, as poorly designed education programs or seminars with little relevance to its target audience will have much less impact than a well-designed program with highly relevant content [15,63].

To overcome potential challenges when holding larger meetings, Brian commented that although he was not sure if it was a feasible option (whether it was due to beliefs about the resourcing and time needed to do this), he thought that having smaller meetings in a more one-on-one environment, that is, at a football club, may be the ideal scenario rather than three leagues combined:

Having such a meeting at separate football clubs might be an option. It’s more personal, and implementation could quite possibly be more successful, because people will feel more comfortable asking questions in a one-on-one environment, than they would in a three-league environment.

(Brian)

Many clubs may value and respect the option of a small group meeting in terms of the provision of personalized information and consultation. It is likely this strategy would also provide the opportunity for people in clubs to speak more freely about their ideas and concerns, and explore strategies and plans of action that could be contextualized to their club. It is unclear at this stage, whether such a smaller group situation at a club is a better strategy or more feasible than a larger public meeting/forum in community-AF contexts. Nevertheless, these strategies could also be used separately or in combination and will need to be explored to evaluate what might work best. For individual club consultations to occur, clubs may need to consider the frequency of meetings or the availability of funding and resources. Further strategies including the opportunity for individual mentoring or other advice may also be useful for some stakeholders, such as the coach, and this could be negotiated.

### 3.6. The Continuing Need for Coach Training Programs

A main theme identified was coach education, learning and development pathways. In a previous study with community-AF coaches [22], it was established that community-AF coaches learn through engaging with a wide range of learning sources in various situations. This is supported in the broader coaching literature [64,65]. When speaking with coaches about strategies for wider diffusion of IPEPs, links with such notions of learning were recognized. All coaches particularly commented about formal Australian Football League Coaches Association (AFLCA) accreditation pathways, and non-formal learning situations as strategies.

#### 3.6.1. Formal Learning

The formal modes of accreditation and reaccreditation pathways were important avenues mentioned by all coaches. According to L. Woodman [66] (personal communication, 19 January 2015) there are about 6000 new Australian Football coaches, together with existing coaches, that complete their reaccreditation per year. There are various types of coaching courses to obtain accreditation and reaccreditation in Australian Football designed to suit specific needs of participants in each of the coaching age groups (junior to high performance/elite), including differing content and formats (for example, attendance at seminars, involvement in training sessions conducted by other coaches, particularly mentor coaches [67]). One coach commented:

There’s hundreds and hundreds of coaches a year, that perform a level one or level two coaching accreditation, where if you can get the program, not authorized, but approved upon, and have the VFL, AFL or league level, or whoever runs the coach accreditations, to support the program (IPEPs). They (the AFLCA) get guest speakers in, and they get football tacticians, they get the support, they get the fitness advisors, or maybe you can get yourself into an hours presentation to a level one or two coaching course accreditation, and then you start to hit the coaches at that level as well. … if you can get them to approve, then you are going to be hitting another level of coaches each year.

(Brian)

It is likely that this suggested strategy has the potential to reach novice coaches stepping into coaching roles especially, as all states and territories in Australia support the AFL’s mandatory coaching accreditation policy. Under this policy, coaches must have completed an approved coaching accreditation course if they are to be appointed to a coaching position. Additionally, reaccreditation and ongoing professional-development processes could be extended to more experienced coaches or previously accredited coaches. Annually, up to 25,000 coaches are registered with the AFLCA and the turnover rate is 25% [66]. Therefore, this suggested strategy may miss some coaches. However, it is one strategy that can be used to promote IPEPs and has the potential to evolve and be enhanced over time to support coach development/skills and reduce the risk of players’ LLIs. Whilst taking such an approach to utilise existing formal or informal learning structures through the AFLCA would appear logical, there may also be inherent barriers or issues within the formal education/accreditation scheme that would need to be taken into consideration and possibly modified or improved (for example, adherence to the mandatory coaching accreditation policy).

Consistent with the coaches’ comments, a first step in reflecting and reviewing key issues would be to consult and collaborate with the AFLCA, state coaching managers, and/or, regional development managers. Secondly, ongoing research and evaluation of coaches’ perceptions and needs surrounding accreditation and content of courses across the board could be undertaken to clarify any similar concerns/issues. There has been little research on how coaching courses are perceived by coaches. Coach educators have indicated that during formal education some key issues are communicated by coaches which could be systematically explored and evaluated [65,68]. Taken together, these strategies allow the added bonus of giving coaches (on a wider scale) a level of ownership about their learning experiences whilst also providing feedback to the AFL bodies that devise coach-development programs to improve their effectiveness and evolution. Thus, while the coaching environment is a place of learning for both coaches and players, coach educators must also be recognised, together with their own developmental and learning needs, to support any issues that may need to be overcome (for example, in coaching contexts), and be able to disseminate pertinent information to facilitate coaches to perform their roles as optimally as possible. The coach educator has a role to play in coach learning, contributing to efforts to raise coaching standards and developing coaching as a profession.

Many coaches adapt innovative learnings or interventions to meet their own needs, and often do so through trial and error [38,69]. In quantitative findings, coaches’ intentions to modify IPEPs align with such notions [22]. Thus, appropriate subject knowledge needs to be developed. However, this still does not presume that coaches are able to apply this knowledge in practice. This potentially raises issues of fidelity [70,71] and it may turn out to be a different intervention through such a process. This is a question that could be debated and tested [72]. As Dearing et al. [55] point out, it is important to understand why a program works and keep these mechanisms and concepts (effect fidelity), while adopters should be able to change peripheral components of a program (program fidelity) so that it constitutes a better fit. Nash and Sproule [73] found that the translation of information presented at a coaching course into readily useable material was debatable. Some coaches found information was easily translated and used, but a number of more experienced coaches recognized that the content needed to be contextualized prior to its use. The study of the mechanisms (or mediators) of behavior change will be critical to understanding this issue of effect fidelity versus program fidelity in the future.

In this study, coaches indicated that informal or more practical coaching experience and observing other coaches are the preferred methods of coach learning. This is consistent with extant literature related to preferred methods of coach learning [64,74,75,76], and needs to be taken into consideration by sport-injury specialists. This supports the comments related to working on implementation processes and, for example, may include provision of ongoing support and mentorship for coaches during implementation.

#### 3.6.2. Non-Formal - Seminars and Workshops

Learning in non-formal situations is well established and is often a preferred learning pathway for many coaches with its implications for knowledge development and professional socialization being recognized in the coaching literature [77,78,79]. Extending the formal links above, all coaches believed that a seminar or workshop would be a valuable way to support IPEPs scale-up and could be something that is integrated on an ongoing basis into coach professional development, similar to the accredited courses. This is supported by Steffen et al. [4] who suggested proper education of coaches during an extensive pre-season workshop was effective in supporting team adherence.

Two of the coaches suggested how a coaches’ forum (seminar or workshop) could be undertaken. They believed the proper timing of the forum, at the end of the year before pre-season starts, would be important. This is reflected in the following:

Well, I think from a coaches’ point of view, just generally speaking, that this sort of thing could be organized … maybe at the end of the year, you know, before pre-season starts as a refresher and going through these sorts of the things (the IPEP) at a coaches’ forum would be fantastic.

(Geoff)

I think it’s just a part of coaching. It’s like going to any coaching seminar, I think. Yeah, so I think they could … Like they do a coaching seminar at the end of every year for new coaches that want to do this level of accreditation (Level 2), so I think that would be a good day to do something like the IPEP … that focuses on the injury sort of stuff as well. To try to work in with that too.

(Andrew)

One of the coaches reiterated that he would feel confident implementing the program himself. However, there was a sense that he believed that many coaches would not have such expertise or experience, and that, being involved in a seminar that was practically based, whilst allowing time for learning and reflection on how they could implement the program, would be useful. Taking this approach was deemed particularly important for coaches to develop training plans and ensuring they had the confidence and behavioral capability to implement IPEPs effectively. He remarked:

I know I can implement these things because I’m still involved in the industry (sport and exercise science) but for a general coach, having them or educating them at the start of the season is too late. I would say probably early November would be perfect because a lot of teams start to train again in about November. So, that would probably be the perfect time because if they are doing it right, they’re setting out (planning) their programs (or training schedules). They get this program (IPEPs), they get some ideas and they’d be able to run with it.

(Geoff)

Ongoing development of coaches and reinforcement approaches to continue the use of IPEPs via refresher courses was also a means one coach felt was important:

So, I think that would be an ongoing one (the forum). They (coaches) just need to get constant reinforcement. If it’s put in front of them as a good idea, and it’s in their face, they will use it. If it’s something that you do every three years, they’ll do it for 6 months, 12 months. The second year and the third year, they’ll forget it. Once they get the refresher … ‘oh yeah that was a good idea, bring it back in’. Obviously, the costs’ there, it’s the personnel just going through it. You’re trying to train up coaches. Or their assistant coaches or staff that they’ve got there. But ongoing development for those people would be fantastic.

(Geoff)

This point restates the belief that reinforcement approaches [37] are important. However, there is also a need to consider available or appropriate resources to facilitate ongoing education. Also highlighted is the need to not only train coaches but others in the football setting that may implement or deliver the program, such as fitness professionals, exercise scientists, or leadership players.

These findings relating to concepts of non-formal (seminar and workshop) learning appear to be an opportune strategy for disseminating IPEPs to coaches. This could be used in parallel with formal pathways and would appear to be an ideal step forward in better supporting the ‘how’ of coaches’ learning. Whilst it is likely that further research into understanding how coaches learn across a wider spectrum of community-AF is needed, it is also likely that movement beyond the identification of coach learning sources and situations will also be needed to consider the complexities, intricacies and nuances that are an inherent part of the learning process.

### 3.7. Policy Drivers

Coaches’ suggested policy as a further strategy that may assist support dissemination and scale-up efforts. Policies are generally defined as formal and informal rules that can guide planning, implementation and evaluation [61]. There are already numerous policies at all levels of AF, though there can be some variability in their use and the types of policies used (for example, formal policies ranging from match play, respect and responsibility, sport trainers, member policies to alcohol and illicit drugs) [80,81,82].

Andrew was optimistic and supportive, providing other examples whereby the injury rehabilitation area has evolved and improved over many years, and the same would be advantageous in relation to injury prevention. He commented:

Well, I’ve seen a lot of changes and it wouldn’t be a bad thing and it would surprise me if it happens. I think it would be good. I would definitely be an advocate for it but … I’ve seen a lot of changes over 10 years where injuries and the improved awareness and that’s great … that we are in much better position than what we were 10 years ago, that’s for sure. Injuries 10 years ago, where a player might never play again and something from a simple knee clean out. Knee rehabs these days–you know, one of the guys in Ballarat had the keyhole surgery on the knee and was back in seven weeks. Like that Sydney guy, Malcheski or whatever his name was, had (new intervention for injury). So, those sort of advances I’ve talked about, and … the potential for policy in injury prevention are great.

(Andrew)

Another coach believed policy could be a good step but he also wondered if there might be some barrier to implementing policy with different leagues having different constitutions:

The policy side of things is always going to be difficult … Because leagues have different constitutions. You need to go through the VCFL, the Metro and Amateurs boards and go through trying to implement it through those people. The AFL overseas everything, they’re obviously the peak body but they delegate quite a lot. For my club, it’s the VCFL. For Essendon districts it’s the Metropolitan Football League. And then you’ve got the amateur association. So, there are three bodies that you’d need to go through.

(Geoff)

This finding suggests that there may be some difficulties in developing a formal policy given different constitutions across league structures. It is likely that the AFL as the peak body would need to be involved largely to support such dissemination. Policy would also need to written with input from a range of people, including club boards, administrators, coaches, players, trainers and other personnel and consider a range of factors in development [82]. It is often the case, as pointed to earlier, that clubs develop their own policies. These informal rules can differ between clubs. Clubs could be encouraged and be provided with additional resources and skills to develop policies to support the selection of coaches in line with the expectations of players. Evidence-based guidelines could also be developed to support the use of injury-prevention policies and be posted on the community-AF website, as a key example. An approach in collaborating with league boards could also be possible. This could occur in stages and at least assist in getting injury prevention on the agenda.

### 3.8. Ensuring Multi-Level and Multi-Dimensional Action

Embedding the program at all levels of AF to ensure sustainability despite any environmental or organizational changes was believed to be important. For example, one coach discussed the key aspects of integrating LL-IPEPs at multi-levels. He supported notions of players reinforcing the program and influencing coaches’ training behavior, club commitment and mentorship support for the coach:

That’s where the—if you had it built into the accreditation process, it will keep going. Once something is in a club, the harder it is to get it out. Each coach that is coming in (that is. a new coach transitioning into a club) might have their ideas, but players will say nuh (no) we do this. I had a number of drills, ‘oh we call it this, we did this process or that’s what we used to do with this other coach’. They know, they remember. Players remember. So, they will all suggest it, and when a club brings in a new coach, generally that coach will have a designated person to help mentor them within the club. So if it’s a part of what is working and the clubs accepted, yes, this is what we want to do, it should not fade out. It’s only when you get those massive whole-board changes; the coaching staff changes, that’s when you might lose the program. But for a general club to club, it may change slightly. Aspects of the program might change or the time given might change slightly but the elements will still be there.

(Geoff)

This finding mirrors increasing recognition among injury researchers of the need for many sport injury-prevention interventions to target multiple levels of sport delivery [3,83]. Implementation strategies, at various levels, as illustrated by the RE-AIM Sport Setting Matrix [35,76] are needed to plan program adoption, implementation and sustainability.

### 3.9. Developing a Backup ‘Hotline’ for Coaches

Having a contact person devoted to injury prevention, or a ‘hotline’, as a support for coaches for the delivery of IPEPs was deemed as an important strategy over the longer term. One coach suggested it as a strategy to engage and increase emphasis on injury prevention:

I think having a contact, some sort of hotline to call would be helpful. If I get a program like that (the IPEPs), first thing I’d say is where has it come from? Who am I going to speak to? If I was going to coach, next year and the year after that, I want to know more. So, who’s the person I can contact? Is there someone, a point of contact or a helpline I can use? Somewhere that I can call, or someone to contact to talk to about at any stage, this would be great.

(Brian)

This finding suggests that such a strategy should increase its emphasis on injury prevention and IPEPs’ maintenance behavior for coaches. Therefore, it would seem important that developing a dedicated role whose primary responsibility would be a contact for injury prevention in community football might be a worthwhile endeavor. Providing support and mentorship for coaches on injury prevention and related sport science is important. Indeed, New Zealand Football (NZF) [7] have taken such an approach and employed a person whose primary responsibility is injury prevention, but who was also available for other duties. This gave the topic of injury prevention credibility and was essential in developing a coalition with NZF. Subsequently, resourcing and budget funds established and expanded the role within NZF. While community-AF may not be in the same position as NZF at this time, it could be possible to create such a role in the future.

### 3.10. Ongoing Evaluation and Feedback Mechanisms

To evaluate the ongoing effectiveness of IPEPs, coaches suggested research investigating the changes in injuries relative to IPEPs and performance over the course of each season would be beneficial. For example, Brian commented:

I think ongoing data attention to injury reporting and other aspects (performance) would be useful and supportive, ongoing collation of data and any extensions to the program would be something I would be looking for … I am one of those coaches who like to evolve and knows what going on.

(Brian)

The need to initiate comprehensive surveillance systems and ongoing evaluation of injury prevention initiatives in community-AF was reported as important. This would appear an important feedback and reinforcement loop [37] for community coaches, in addition, to supporting ownership and strengthening community-AF in continued efforts to deliver quality IPEPS. Additionally, as new research and best-practice injury prevention evolves and accumulates, appropriate modifications to IPEPs can be made. Models and schemes in other sport settings have successfully been applied and shown promise in increasing the safety and competitive equity for a range of athletes of all ages and levels [7,84]. Ongoing evaluation and dissemination of information on injury in community-AF could justify continued investment in existing IPEPs, as well as in other sport-injury intervention areas that warrant investigation in the future [16,23,85].

## 4. Conclusions

This is the first qualitative multiple case study to explore community-AF coach perspectives about the nature and range of strategies that could be used to maximize dissemination and scale-up of IPEPs in order to prevent LLIs. The information gained from this research provides valuable insights into the scale-up of interventions and the needs of coaches from a coach’s perspective. This can provide guidance to researchers and applied consultants on dissemination and scale-up in community-AF contexts. The strategies that coaches considered are aligned with previous or current strategies to promote the prevention of health and other injury conditions [39,61,84,86]. There is some evidence, in the extant literature, to support the critical role of strategies identified by coaches, albeit with challenges, and ideally sport injury-prevention efforts should build on the coaches’ views and enhance the strategies that have been tried and found effective [13,20,84,87,88,89,90,91,92,93,94]. As such, information obtained from coaches can be used to form part of the broad backbone of injury-prevention research and practice agendas in Australian Football [23].

While the strategies throughout this study have been discussed separately with some interlinkages provided, they should be understood as interconnected modes of a complex process rather than discrete entities. In reality, many of these strategies could exist in concert or conflict. It is also recognized that coaches acquiring information or learning in one situation will influence a coach’s engagement in other situations [95,96]. Thus, in an effort to promote opportunities for community-AF coaches, and develop a community of learning in injury prevention, a range of strategies could be applied. Research into effective dissemination and scale-up strategies in sport-injury prevention is still relatively sparse and more is needed. In particular, greater research is needed to develop, test and evaluate the impact and outcomes of a range of strategies targeted at coaches and other stakeholders in various sport settings.

A central premise of this latter point and the findings in this study is that injury-prevention improvements require an understanding of multi-level determinants of coaches’ (and that of others) health and injury prevention behaviors, and a range of change strategies at the individual, interpersonal and macro levels is required [83]. Furthermore, the view that societal-level changes and supportive environments are necessary to address injury problems successfully and to maintain individual-level behavior changes seems to be widely endorsed by coaches. This study builds on the intrapersonal and interpersonal theories and associated determinate findings to explain and affect community change, clearly emphasizing an ecological systems multi-level perspective.

While some of the strategies mentioned are already being implemented in AF for other purposes (e.g. [97,98,99]), these strategies could be mapped to fully understand the current AF context as well as existing and new ways to foster and integrate injury prevention together with other priorities. Including injury prevention as a key component in coach development is critical. This can support continued efforts to develop and prepare coaches to facilitate safe and sustainable participation and performance for their players as part of their roles.

Although delivering new injury-prevention programs into community-AF has numerous added benefits, its wider impacts at the individual, team, club and community levels should be recognized [100,101,102]. Whilst each potential strategy, its nature, and effectiveness was not explored in detail with coaches, interviewing and understanding coaches’ views provided the opportunity to collaborate with coaches. The interview discussions with coaches also afforded an understanding of what strategies coaches perceived would be useful and had the promise to tap into the wider community-AF coach community. In addition, the potential to optimize multi-levels of influence and action was highlighted. Even though the scale-up of effective intervention measures under real conditions may prove to be an ongoing challenge [3,7,11,60,69,103,104,105,106], future research could tap into such multi-strategies suggested by coaches. This would optimize monitoring and evaluation efforts in practical and creative ways in an attempt to bridge implementation and sustainability gaps. Integration of group-, organizational-, and community-intervention frameworks with individual and interpersonal models of injury-prevention behavior could optimize the impact and exceed the use of any one approach [3,84]. Advances in research will add clarity to mechanisms of the theories and models of operation and refine understanding on how best to use them. Injury prevention and associated behavior-change strategies will achieve greater success through the application of these frameworks for social activation and community attitude and behavior change [3,82,96,107].

The results of this study provide further evidence for practice frameworks and align with long-term visions to continue assessing evidence- and practice-based interventions in sport internationally [83]. Particular attention in seeking cooperation, and working towards consensus with key stakeholders to adopt IPEPs to prevent LLIs, is important to ensure initial success. Multi-level strategies that can be comprehensively analyzed and described are also vital. Planning actions to support wider coach uptake of IPEP interventions often requires different approaches (for example, related to the content of workshops or seminars, social marketing). Therefore, modification of strategies promoted at wider coaching audiences may be needed to maximize coach effectiveness and skills, such as interpersonal skills. These and other key aspects, for example, the exploration of a coach’s scope of practice in community-AF, together with current evidence, will likely guide ready responses to challenges ahead.

IPEPs, no matter how effective, cannot enhance public health unless they are adopted and maintained in an appropriate and timely manner by coaches and community-AF clubs. Currently, few evidenced-based IPEPs have been systematically and rigorously disseminated and evaluated in community practice. This study addressed this significant research-to-practice gap. If the study objectives and findings are successfully adhered to in the future, a stronger theoretical basis and understanding of effective strategies aimed at implementing IPEPs in real-world contexts can be achieved in order to support the prevention of LLIs…
